# Surface energy-directed assembly of liquid metal particles for intelligent wearable electronics

**DOI:** 10.1016/j.isci.2026.117629

**Published:** 2026-09-25

**Authors:** Yubing Liu, Siqing Yuan, Kaiyi Xu, Linquan Jiang, Zhimin Chai, Xinchun Lu

**Affiliations:** 1 Beijing Key Laboratory of Atomic-Scale Precision Manufacturing and Cross-Scale Device Fabrication, Department of Mechanical Engineering, Tsinghua University, Beijing 100084, China; 2 State Key Laboratory of Tribology in Advanced Equipment, Tsinghua University, Beijing 100084, China; 3 Weiyang College, Tsinghua University, Beijing 100084, China; 4 College of Business, Beijing Union University, Beijing 100025, China

**Keywords:** liquid metal, directed assembly, surface energy, strain sensors, electrode arrays

## Abstract

Liquid metals (LMs) are promising for flexible electronics owing to their high electrical conductivity and inherent room temperature deformability. However, their high surface tension poses major technical barriers for high-resolution patterning. While conventional pre-patterned metal seeding layers can enable deterministic LM patterning, this approach inevitably adds redundant fabrication steps and increases process complexity. Here, we develop a seeding-layer-free surface energy-directed assembly process based on ultrasonically dispersed, sodium alginate-stabilized LM particle dispersions, which enables high-precision LM patterning on various stretchable substrates. The fabricated strain sensors exhibit <200 ms response and >1,000-cycle stability, while the LM electrode arrays paired with a convolutional neural network achieve 94% accuracy in gesture recognition. This work establishes a high-precision, environmentally benign, and broadly applicable platform for next-generation LM-based flexible and stretchable electronics.

## Highlights

• Sodium alginate stabilizes liquid metal particle dispersions effectively
• Surface energy-directed assembly enables non-seeding-layer liquid metal patterning
• Liquid metal-based strain sensors feature rapid response and cyclic stability
• Liquid metal arrays with deep learning achieve 94% gesture classification accuracy

## Graphical abstract

**Figure:**
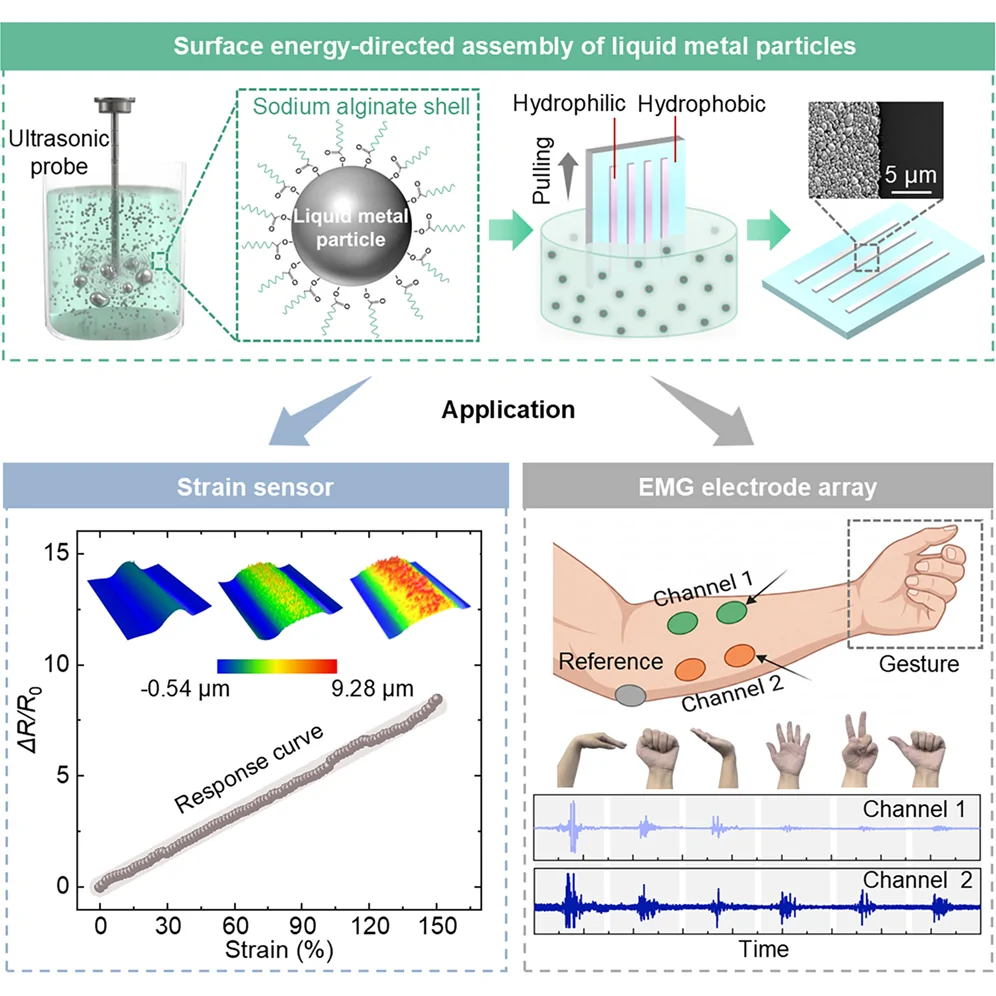

## Teaser

Bioelectronics; Devices

## Introduction

The rapid development of smart displays,^1^^,^^2^ soft robotics,^3^^,^^4^^,^^5^ electronic skins,^6^^,^^7^^,^^8^^,^^9^ and wearable health monitoring systems^10^^,^^11^^,^^12^ has imposed urgent requirements on next-generation conductive materials that possess both high electrical conductivity and stretchability. Although traditional rigid metal films can achieve a certain degree of stretchability through serpentine or three-dimensional helical structure designs,^13^^,^^14^ their complex geometric patterning processes limit scalable manufacturing. The strategy of embedding conductive nanomaterials, such as silver nanowires and carbon nanotubes, into elastomer matrices can provide exceptional stretchability.^15^^,^^16^^,^^17^ However, the irreversible destruction of conductive networks during stretching often leads to the degradation of electrical performance, and the electrical conductivity of these materials is generally insufficient to meet the demands of high-performance devices. Liquid metals (LMs), particularly eutectic gallium-indium (EGaIn) and gallium-indium-tin alloys have emerged as ideal candidate materials for flexible and stretchable electronics, due to their room-temperature fluidity,^18^^,^^19^^,^^20^^,^^21^ high electrical conductivity,^22^^,^^23^^,^^24^^,^^25^ and biocompatibility.^26^^,^^27^^,^^28^

Recently, researchers have developed various LM patterning strategies, among which printing methods stand out as one of the most extensively explored routes.^29^^,^^30^ In screen printing, for instance, copper-doped EGaIn has been formulated into paste for pattern deposition.^31^ However, their resolution is restricted by the screen mesh size, which typically falls within the range of hundreds of micrometers, and the intense shear forces generated during printing can induce ink degradation and subsequent performance decline.^32^^,^^33^^,^^34^ Direct writing offers a mask-free alternative.^35^^,^^36^ As a representative example, natural polyphenol oligomeric proanthocyanidins have been employed to modify EGaIn particles for multi-substrate direct writing.^37^ Nevertheless, the linewidth achieved via this method remains fundamentally limited by the nozzle diameter, and precise regulation of processing parameters is mandatory to guarantee deposition uniformity.^38^^,^^39^ In general, these printing-based approaches are plagued by insufficient patterning precision.^40^^,^^41^ Recently, the surface energy-directed assembly (SEDA) process has emerged as a compelling strategy to break these precision limitations, enabling linewidths as low as 800 nm.^42^^,^^43^ The core principle of SEDA lies in pre-patterning high-surface-energy regions on low-surface-energy background substrates, where functional materials selectively deposit through targeted solution wetting on the predefined patterns and spontaneous dewetting from the non-patterned regions. However, bulk LM exhibits an extremely high surface tension (625 mN m^–1^), which makes it prone to form isolated droplets on the patterned regions rather than spontaneously wetting the entire patterns.^44^^,^^45^^,^^46^ To overcome this formidable surface tension barrier, high-surface-energy metals such as copper and silver are introduced as seeding layers to facilitate LM wetting. In most prior studies, metal patterns are fabricated on substrates through the integrated workflow of photolithography, evaporation/electroplating, and wet etching.^47^^,^^48^^,^^49^ While this approach delivers high resolution with linewidths below 20 μm, the preparation of these metal patterns heavily relies on sophisticated micro- and nanofabrication equipment, leading to intricate processing steps and prohibitive costs. In our previous works, we have exploited the SEDA process to fabricate silver nanoparticle-based patterns as seeding layers for LM wetting and successfully achieved high-quality LM patterns.^50^ However, although complex film deposition and etching processes are circumvented, the mandatory requirement for metal seeding layers still complicates the overall fabrication workflow. To eliminate the need for metal seed layers entirely, it is imperative to break the inherent high surface tension constraint of bulk LM. Fragmenting bulk LM into micro/nanoscale particles to form homogeneous liquid suspensions represents an effective paradigm,^51^^,^^52^ where the surface tension of the resulting LM suspensions is predominantly governed by the dispersing solvents. Consequently, LM particles in the suspensions can be assembled via the SEDA process in a manner analogous to that of conventional nanoparticle dispersions.^53^^,^^54^ However, since LM particles dispersed in solution are prone to severe aggregation and rapid sedimentation due to their relatively large size ranging from hundreds of nanometers to several micrometers,^55^^,^^56^ the critical challenge lies in formulating stable LM dispersions that can maintain long-term dispersion stability throughout the entire SEDA process.

In this study, we report the fabrication of LM patterns without pre-patterned metal seeding layers, enabled by an aqueous LM dispersion stabilized by sodium alginate (SA) molecules and the SEDA process. During dispersion formulation, SA molecules anchor onto the particle surface via coordination interactions between their carboxyl groups and the native gallium oxide layer on LM, forming a conformal protective thin film. The dissociation of sodium carboxylate groups (−COONa) from SA imparts uniform negative surface charges to LM particles, which endows the resulting dispersion with exceptional long-term dispersion stability. Subsequently, the SEDA process is implemented to achieve LM patterning with high fidelity on a broad spectrum of flexible substrates. Leveraging this technique, flexible and stretchable sensors are fabricated by constructing LM conductive lines with tunable widths, whose sensitivity, response time, cyclic stability, and human motion-capturing capability are systematically characterized. Furthermore, electromyography (EMG) electrode arrays based on LM patterns are fabricated. Integrated with a convolutional neural network (CNN), these arrays enable highly accurate gesture recognition, which opens a new pathway for the application of LMs in human-machine interfaces and wearable electronics.

## Results

### Preparation and characterization of the SA-stabilized aqueous LM dispersion

The stable dispersion of LM particles in aqueous media constitutes the fundamental prerequisite for the SEDA process. As schematically illustrated in Figure 1A, the preparation of the aqueous LM dispersion employs a straightforward probe sonication method. EGaIn alloy is mixed with pre-prepared aqueous SA solution and subjected to continuous ultrasonic treatment, during which the localized high temperature and intense shear forces induced by ultrasonic cavitation fragment bulk LM into spherical particles spanning from micrometer to nanometer scale. As depicted in Figure 1B, dissolved SA molecules spontaneously anchor onto the freshly generated particle surfaces to form a thin, dense protective film. This interfacial assembly process is driven by the coordination interaction between exposed −COO^−^ groups on SA molecular chains and the rapidly formed native gallium oxide layer (Ga_2_O_3_) on the LM surface. In Figure 1C, the morphology of the as-sonicated LM particles is further characterized by transmission electron microscopy (TEM). The LM particles maintain a well-defined spherical geometry after ultrasonic dispersion, with their surfaces conformally coated by a uniform, continuous SA thin layer with a measured thickness of approximately 20 nm. As validated by the energy-dispersive X-ray spectroscopy (EDS) elemental mapping in Figure 1D, the signal of In, which serves as a characteristic element of LM, is concentrated in the core region of individual particles. In contrast, Ga is homogeneously distributed throughout the particle core while simultaneously exhibiting a distinct ring-like enrichment in the peripheral region. Similarly, O presents an enhanced distribution at the periphery. This characteristic core-shell elemental distribution pattern directly corroborates the partial surface oxidation of LM particles and the uniform encapsulation of SA on their outer surfaces. According to the scanning electron microscopy (SEM) results presented in Figures 1E and 1F, systematic particle size distribution statistics are further performed. After 30 min of sonication, the LM particles exhibit a broad size distribution ranging from approximately 100 to 1.8 μm, with the highest population centered at 500 nm. As the sonication duration progresses to 60 min, the particle size distribution shifts toward a smaller dimension, with the majority of particles centered at approximately 250 nm (Figure S1). When the sonication duration is extended to 90 min, the size distribution narrows to approximately 20–550 nm, with the majority of particles concentrated at 100 nm. These results quantitatively demonstrate that prolonged sonication can further fragment LM particles and substantially reduce the average particle size.

**Figure 1:**
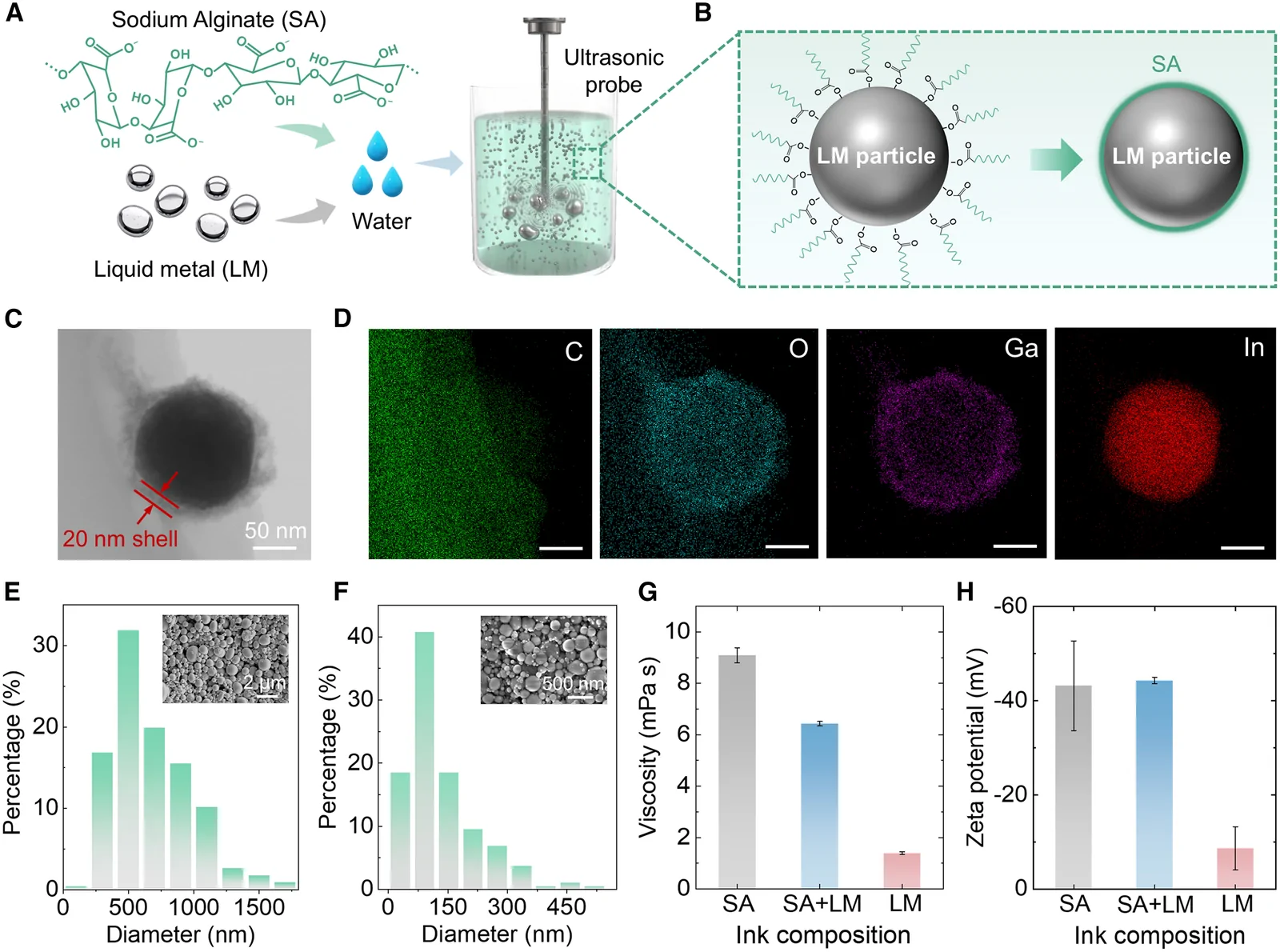
Preparation and characterization of the SA-stabilized aqueous LM dispersion (A) Schematic illustration showing the LM dispersion preparation via probe sonication. (B) Schematic illustration showing the formation of a uniform SA shell on the surface of LM particles. (C) TEM image of one SA-coated LM particle, revealing the distinct core-shell geometry. Scale bar, 50 nm. (D) EDS elemental mapping of C, O, Ga, and In, which verifies the core-shell structure of the SA-coated LM particles. Scale bars, 50 nm. (E) Statistical particle size distributions of LM dispersions after 30 min of sonication. Scale bar, 2 μm. (F) Statistical particle size distributions of LM dispersions after 90 min of sonication. Scale bar, 500 nm. (G and H) Quantitative comparison of viscosity values measured at a shear rate of 1 s^-1^ (G) and Zeta potential values (H) among three samples: pure SA aqueous solution, SA-modified LM particle dispersion, and bare LM dispersion without SA additives. Data are represented as mean ± SD (*n* = 3).

As plotted in Figure 1G, the bare LM aqueous dispersion without SA addition exhibits a low viscosity of 1.39 mPa s at a shear rate of 1 s^−1^. In comparison, the SA-stabilized LM dispersion delivers a measured viscosity of 6.43 mPa s, which is approximately 4.6 times higher than that of the bare LM dispersion. To quantitatively assess the long-term colloidal stability of the as-prepared dispersion, Zeta potential measurements are conducted, with results summarized in Figure 1H. The bare LM particles dispersed in deionized water show an absolute Zeta potential of merely 7.9 mV, which falls within the rapid agglomeration regime defined by classic colloid chemistry theories.^57^^,^^58^ This result is in excellent agreement with the experimental observation that bare LM particles undergo rapid sedimentation within a short period (Figure S2). Conversely, the pure SA aqueous solution presents a high absolute Zeta potential of 58.4 mV, originating from the dissociation of −COONa groups on SA molecular chains into negatively charged −COO^−^ moieties, which endows the polymer chains with strong negative surface charge. Notably, the absolute Zeta potential of SA-modified LM particles reaches 62 mV, a magnitude nearly equivalent to that of the pure SA solution. This result confirms that the SA coating fully covers the LM particle surfaces, which introduces intense electrostatic repulsion between adjacent particles and drastically enhances the anti-agglomeration stability of the LM dispersion.

### Fabrication of LM patterns utilizing the SEDA process and pattern characterization

This study further investigates the fabrication of LM patterns using the SEDA process. As illustrated in Figure 2A, the SEDA process consists of three sequential stages: selective hydrophilic modification of a hydrophobic substrate, assembly of the LM particle dispersion, and subsequent solvent evaporation from the assembled LM dispersion. During the hydrophilic modification, high-energy ultraviolet (UV) light is utilized to break the polymer molecular chains, generating polar functional groups such as hydroxyl groups (−OH) within the irradiated regions, thereby converting them from intrinsically hydrophobic to hydrophilic. Figures 2B and 2C present static contact angle measurements of three test liquids—pure aqueous SA solution, SA-modified LM dispersion, and bare LM dispersion without SA additives—on polydimethylsiloxane (PDMS) substrates before and after UV treatment. On untreated hydrophobic surfaces, the contact angles of the three liquids are 101.3°, 101.0°, and 112.3°, respectively, all of which exhibit clear hydrophobic behavior. In contrast, on UV-treated hydrophilic surfaces, the contact angles decrease sharply to approximately 6.9°, 5.8°, and 7.4°, respectively, demonstrating superhydrophilic characteristics. During the assembly step, the LM dispersion preferentially wets the hydrophilic-patterned regions while spontaneously dewetting from the hydrophobic non-patterned areas, thereby forming patterned LM dispersion on the substrate. Subsequent evaporation of water from the assembled dispersion leads to deposition of LM particles onto the hydrophilic pattern regions, ultimately yielding LM particles-based patterns.

**Figure 2:**
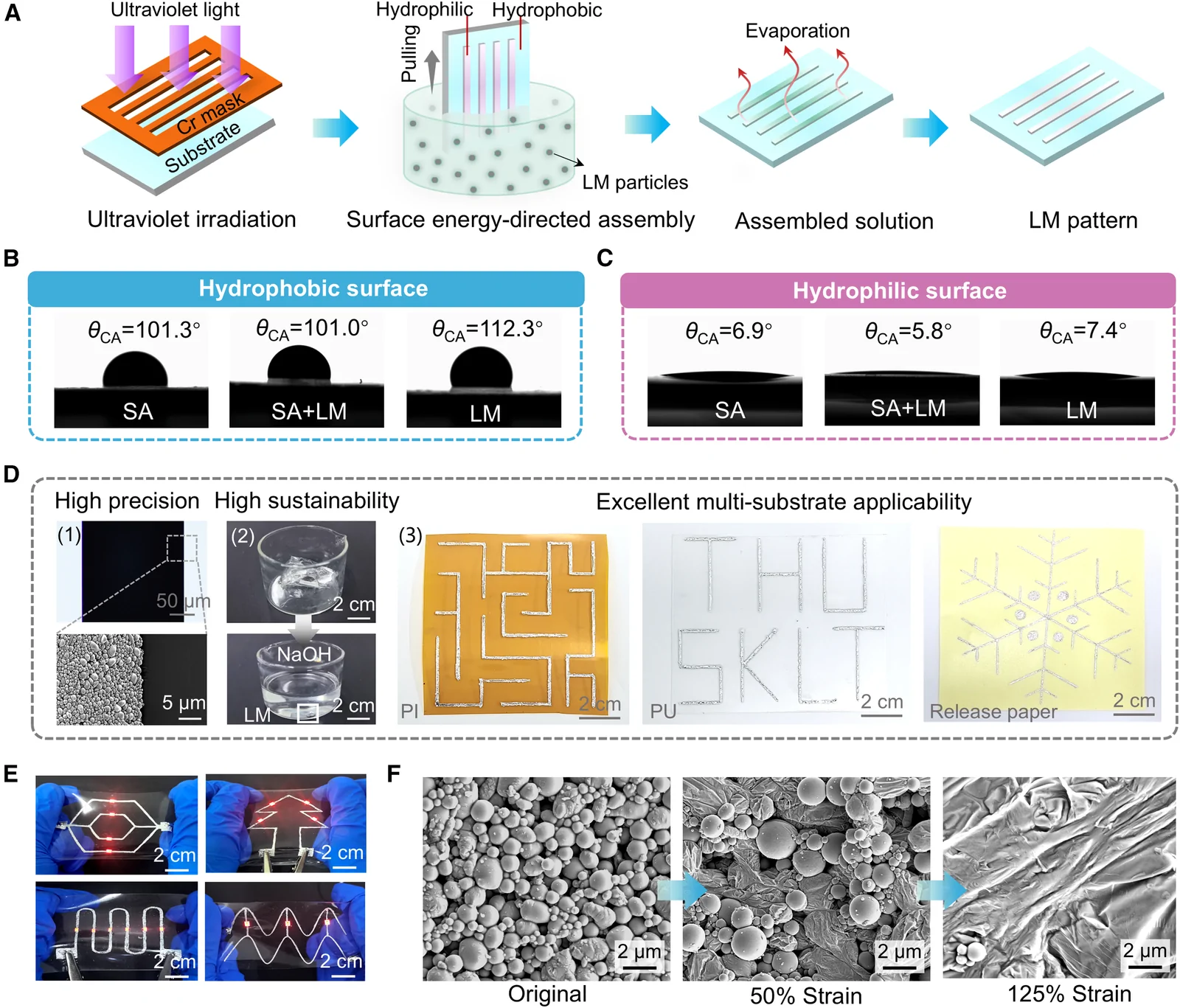
Fabrication of LM patterns utilizing the SEDA process and pattern characterization (A) Schematic illustration of the fabrication of LM patterns using the SEDA process. A substrate with pre-defined patterned hydrophilic regions and hydrophobic background is pulled out from an LM dispersion. During this process, LM dispersion is entrained selectively onto the hydrophilic pattern regions. Upon complete evaporation of the entrained aqueous solvent, patterns composed of densely packed LM particles are formed. (B and C) Static contact angles of different liquids on PDMS substrates before (B) and after (C) UV irradiation. (D) Core advantages of the SEDA process for LM patterning: (D_1_) high precision, (D_2_) high sustainability, and (D_3_) excellent multi-substrate applicability. Scale bars: 50 μm (top) and 5 μm (bottom) in (D_1_), 2 cm in (D_2_), and 2 cm in (D_3_). (E) Demonstration of the high electrical conductivity of the LM patterns under stretching and deformation. Scale bars, 2 cm. (F) SEM images of morphological evolution of the LM patterns during stretching under uniaxial tensile strains of 0%, 50%, and 125%. Scale bars, 2 μm.

Figure 2D demonstrates the three core advantages of the SEDA process for LM patterning. Figure 2D_1_ illustrates its superiority in resolution and edge sharpness. Distinct from conventional solution-processing methodologies such as inkjet printing and screen printing, which are commonly plagued by jagged edge artifacts, the LM lines fabricated via the SEDA process exhibit steep edge profiles and exceptional pattern fidelity. To quantitatively evaluate the patterning resolution of this method, we systematically reduced the width of the predefined hydrophilic patterns. As demonstrated in Figure S3, the SEDA process is capable of achieving continuous LM lines with a minimum width of 200 μm. Furthermore, despite the transfer of a superficial layer to the adhesive tape, the LM pattern remains intact on the PDMS substrate, indicating robust interfacial adhesion (Figure S4). Figure 2D_2_ demonstrates the recyclability of the assembled LM particles. By immersing the substrate decorated with pre-formed LM patterns in a 1 mol/L NaOH solution for several seconds under mild agitation, recycled and agglomerated bulk LM can be collected at the bottom of the container. During this process, the alkaline solution dissolves the LM particle’s surface gallium oxide layer, which drives the bare LM droplets to spontaneously coalesce into macroscopic bulk LM to minimize surface energy.^59^^,^^60^^,^^61^ Consequently, this recycled bulk LM can serve as the initial raw material for a new round of ultrasonic dispersion. This recyclability not only lowers material costs but also aligns with the principles of green chemistry and sustainable manufacturing. As presented in Figure 2D_3_, since the UV-induced surface hydrophilic modification is compatible with a broad range of polymer materials, the SEDA process enables LM patterning on diverse substrates, including polyimide (PI), polyurethane (PU), PDMS, and even paper.

Figure 2E reveals that the intrinsic fluidity of LM endows the resulting patterns with the capability to maintain continuous electrical connectivity under mechanical stretching. Here, LM circuits are fabricated on PDMS substrates to connect multiple light emitting diode (LED) bulbs in series or parallel configurations. When the substrate undergoes tensile deformation, all LEDs retain constant brightness without flickering or extinguishing, despite the geometric distortion of the LM conductive traces. It is noteworthy that owing to the presence of a native oxide layer surrounding the LM particles, the as-assembled LM patterns exhibit relatively low electrical conductivity. The LM patterns require mechanical activation to rupture the insulating oxide layer for the establishment of high-conductivity pathways.^62^ Figure 2F illustrates the SEM morphological evolution of the LM patterns during stretching-mediated activation. At the initial unstretched state, the LM particles are mutually isolated with well-defined outlines. Upon application of 50% tensile strain, the oxide shells of LM particles commence rupturing under mechanical stress. The internal bulk LM leaks out through the interparticle gaps and preliminarily bridges adjacent particles, while a fraction of microspheres still retains their spherical morphology. When the tensile strain is further increased to 125%, all particles are fully ruptured and the encapsulated internal LM is extruded outward. After stretching activation, the LM patterns become highly conductive. Once activated, the LM patterns can maintain stable electrical connectivity under large deformations, rendering them highly promising for widespread applications in fields such as stretchable displays, flexible circuits, and wearable electronics.

### LM line patterns for stretchable strain sensors

Leveraging the SEDA process, LM line patterns with widths of 0.5, 1, and 1.5 mm are fabricated to construct stretchable strain sensors. The three-dimensional morphology of the LM patterns is characterized using white light interferometry, as presented in Figures 3A, 3E, and 3I. From the extracted cross-sectional profiles of the lines (Figures 3B, 3F, and 3J), the thicknesses of the LM patterns corresponding to the 0.5, 1.0, and 1.5 mm linewidths are approximately 1.63, 4.34, and 6.41 μm, respectively, exhibiting a monotonically increasing trend with linewidth. In addition, the cross-sectional profiles of the lines follow a cap-shaped distribution, where the central region is slightly thicker and the marginal areas are relatively thinner. Benefiting from the increased pattern thickness, the initial resistance of these sensors decreases progressively as the linewidth increases (Figure S5). To prevent the continuous oxidation of exposed LM particles in ambient air, which disrupts conductive pathways and significantly increases electrical resistance, the LM patterns are encapsulated with a PDMS layer that effectively isolates the LM from environmental degradation and ensures long-term performance stability (Figure S6). Systematic electromechanical performance tests are performed on the three as-fabricated strain sensors. The resistance response curves in Figures 3C, 3G, and 3K demonstrate that the resistance of all sensors increases monotonically with applied tensile strain. However, the magnitude of resistance increment is moderate due to the high intrinsic fluidity of LM. Moreover, the increment amplitude is comparable across sensors with different LM pattern widths. As a consequence, LM patterns with distinct linewidths deliver similar sensitivity, as quantified by the gauge factor (GF). Subsequently, the response and recovery times of the sensors are quantitatively evaluated (Figures 3D, 3H, and 3L). The results reveal that the response times of all three sensors are within 120 ms, and their recovery times are within 200 ms. Such rapid response and recovery dynamics guarantee the superior dynamic performance of the sensors for capturing high-frequency motion signals. Furthermore, the long-term cycling stability of these sensors is assessed in Figures S7–S9. After applying more than one thousand cycles of repeated loading and unloading, the initial resistances of these sensors increase gradually. This phenomenon is attributed to the progressive oxidation of LM particles during prolonged cyclic stretching, which partially suppresses their intrinsic fluidity. Nevertheless, the waveform and amplitude of the overall resistance response curve remain nearly unchanged, further verifying the long-term operational reliability of the LM-based flexible strain sensors for extended practical use.

**Figure 3:**
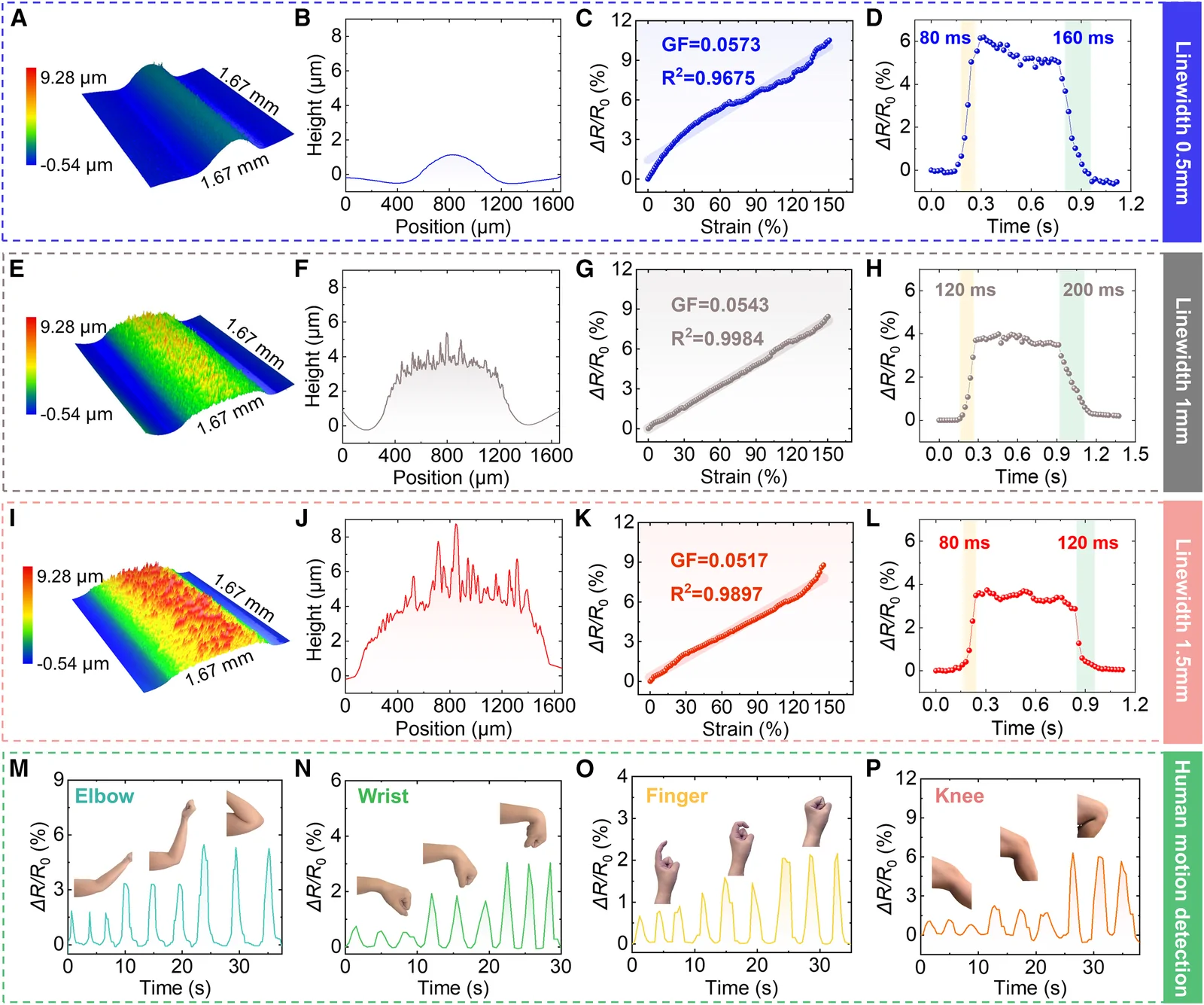
LM line patterns for stretchable strain sensors (A–D) 3D morphology (A), cross-sectional height profile (B), resistance response curve under increasing strain (C), and response and recovery curve (D) for LM sensors with linewidth of 0.5 mm. (E–H) 3D morphology (E), cross-sectional height profile (F), resistance response curve under increasing strain (G), and response and recovery curve (H) for LM sensors with linewidth of 1 mm. (I–L) 3D morphology (I), cross-sectional height profile (J), resistance response curve under increasing strain (K), and response and recovery curve (L) for LM sensors with linewidth of 1.5 mm. (M–P) Real-time resistance response curves for monitoring bending motions of the elbow (M), the wrist (N), the finger (O), and the knee (P).

To validate the human motion signal acquisition capability, the strain sensor with a 1 mm linewidth is conformably attached to the elbow, wrist, index finger joint, and knee. As shown in Figure S10, the fabricated PDMS/LM strain sensor exhibits excellent flexibility and wearability, and can be seamlessly attached to various human joints. During the testing procedure, three distinct bending angles are applied to each joint. As illustrated in Figures 3M–3P, the sensor generates resistance signals with distinctly differentiated amplitudes corresponding to different motion ranges of each joint. Upon the joints returning to their initial unbent configurations, the resistance signals immediately recover to values close to the baseline. These results demonstrate the great application potential of the as-fabricated LM sensors in human motion capturing and wearable healthcare monitoring.

### LM electrode pattern for EMG signal collection

Beyond the application of flexible strain sensors, LM electrode arrays are assembled on PU substrates to verify the adaptability of the SEDA-fabricated LM patterns for EMG signal acquisition. Figure 4A presents the full workflow of signal acquisition, deep learning network processing, and gesture classification recognition. Two independent acquisition channels are established in this LM electrode array, where the elbow with relatively sparse muscle groups is selected as the reference electrode. Two channels of voltage signals recorded from the forearm muscles during movements are collected and wirelessly transmitted via bluetooth to a CNN algorithm deployed on a Python platform. This network consists of an input layer, two convolutional layers, a pooling layer, a flattening layer, and a final output layer. As illustrated in Figures 4B, six gesture labels are predefined, including palm leaning back, making a fist, palm leaning forward, opening palm, victory sign, and thumb extension. The two-channel signals acquired by the LM electrodes exhibit characteristic waveforms highly correlated with specific gesture types, which provide high-quality raw data for subsequent pattern recognition tasks. Subsequently, Figures 4C and 4D systematically compare the signal-to-noise ratios (SNRs) of the LM electrodes and commercial medical Ag/AgCl electrodes under identical experimental conditions. For all six predefined gestures, the SNRs measured by the LM electrodes in both channels are significantly higher than those of the Ag/AgCl electrodes. This performance advantage originates from the room temperature fluidity of LM, which enables the material to penetrate the microscopic depressions of skin texture to form large-area conformal contact. In sharp contrast, the contact area between rigid Ag/AgCl electrodes and the skin is geometrically limited, and inevitable microscopic air gaps may exist at the electrode-skin interface.

**Figure 4:**
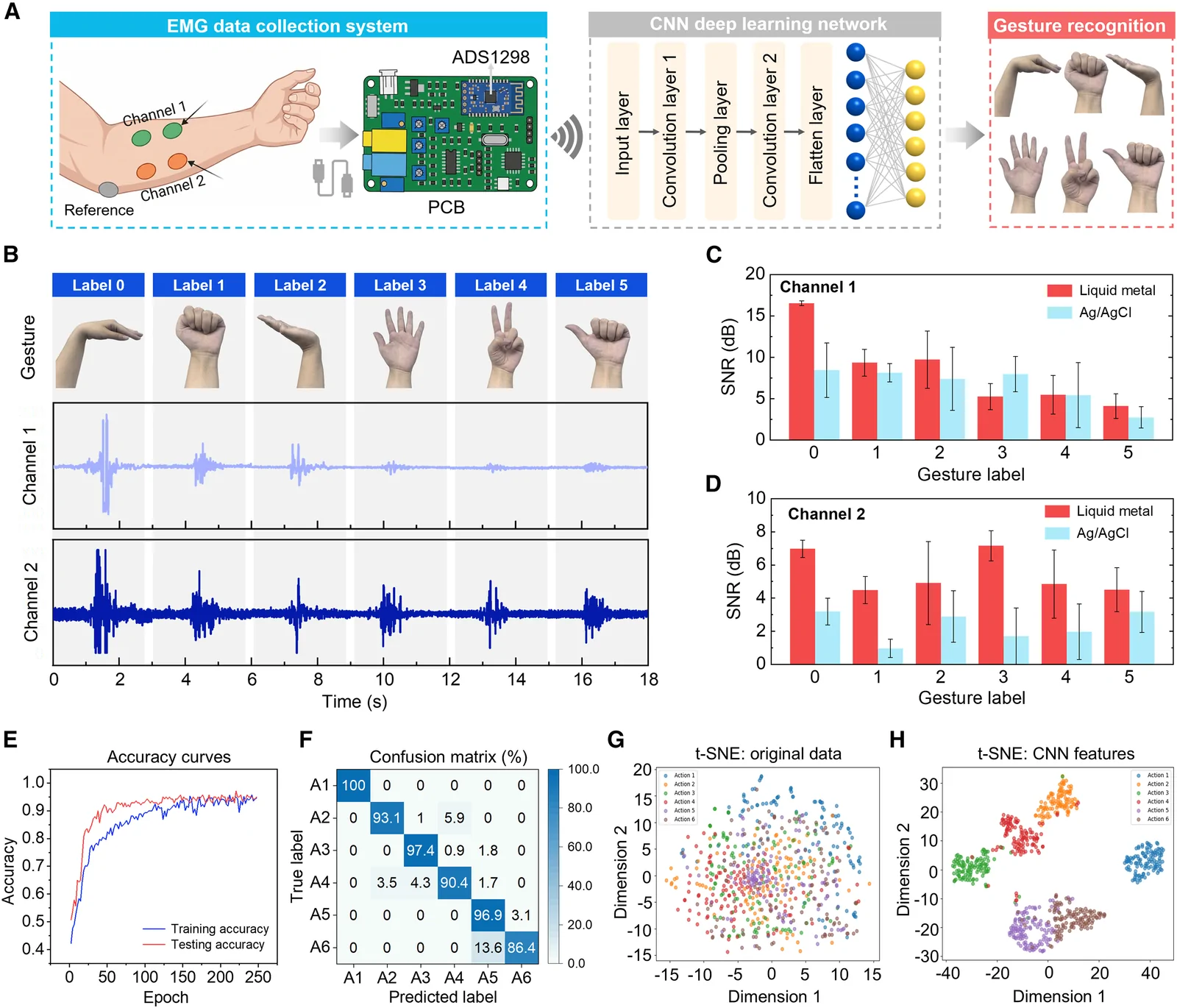
LM electrode pattern for EMG signal collection (A) Flowchart of the two-channel EMG signal acquisition and the CNN-based gesture classification system. (B) Characteristic voltage waveforms acquired for six different gestures. (C and D) SNR comparisons between LM electrodes and Ag/AgCl electrodes in channel 1 (C) and channel 2 (D). Data are represented as mean ± SD (*n* = 5). (E) Training and testing accuracy curves over 120 epochs. (F) Confusion matrix for the classification of six gestures showing average 94% accuracy. (G and H) T-SNE distribution plots of the original dataset (G), and the CNN-extracted features (H).

Next, 600 sets of data are collected for each gesture, where 80% of the total dataset is allocated to the training set and the remaining 20% is assigned to the testing set. After 250 training epochs, both the training and testing accuracy curves asymptotically approach 1 (Figure 4E), while the training loss curve approaches 0 (Figure S11). This result confirms that the network has achieved full convergence and possesses reliable generalization capability. As visualized by the confusion matrix in Figure 4F, the classification accuracy of the testing set reaches 94%. The occasional misclassification of certain gestures arises from the partial overlap of the forearm muscle groups activated during these specific movements. As illustrated in Figure 4G, in the t-distributed stochastic neighbor embedding (t-SNE) distribution plot of the raw dataset without CNN training, data points corresponding to different labels are heavily mixed and overlapped. However, after feature extraction through the well-trained CNN, data points belonging to each label form independent clusters and can be clearly distinguished from those of other categories (Figure 4H). This observation demonstrates that the designed CNN can effectively extract discriminative data features and achieve highly efficient gesture classification.

## Discussion

In this study, the fabrication of LM patterns without pre-patterned metal seeding layers is demonstrated, leveraging an aqueous LM dispersion stabilized by SA and the SEDA process. Stable LM aqueous dispersion is achieved through the anchoring of SA molecules onto the native gallium oxide layer encapsulating LM particles, wherein the dissociation of −COONa groups into −COO- ions imparts a strong negative surface charge, enabling exceptional colloidal stability via electrostatic repulsion. Through SEDA, high-fidelity LM patterns are reliably achieved across various stretchable substrates, including PI, PU, PDMS, and release paper. Stretchable strain sensors are constructed using LM line patterns of tunable widths, exhibiting rapid response times of less than 200 ms, long-term cyclic stability over 1,000 cycles, and the ability to accurately resolve human joint kinematics. Furthermore, LM electrode arrays are prepared onto PU substrates to acquire EMG signals from the human arm. When coupled with a CNN deep learning algorithm, these arrays classify six gestures with 94% accuracy, while achieving SNRs superior to those of commercial Ag/AgCl electrodes. This work establishes a high-precision, environmentally benign, and broadly applicable platform for next-generation LM-based flexible and stretchable electronics, wearable health monitors, and intelligent human-machine interactions.

### Limitations of the study

Currently, the minimum continuous LM line achieved via the proposed SEDA technology is 200 μm. For scenarios requiring higher device integration density, the patterning resolution needs to be further extended toward finer dimensions. Future work may focus on modulating withdrawal dynamics and dispersion properties to expand the high-resolution patterning capability of this strategy, supporting the development of higher integration LM flexible electronic systems.

## Resource availability

### Lead contact

Requests for further information and resources should be directed to and will be fulfilled by the lead contact, Zhimin Chai (chaizhimin@mail.tsinghua.edu.cn).

### Materials availability

This study did not generate new unique reagents.

### Data and code availability

• All data reported in this paper will be shared by the lead contact upon request.
• This paper does not report original code.
• Any additional information required to reanalyze the data reported in this paper is available from the lead contact upon request.

## Acknowledgments

This work was supported by the 10.13039/501100001809National Natural Science Foundation of China (grant no. 52205596), the start-up funding from 10.13039/501100004147Tsinghua University in China (grant nos. 533306002 and 042006001), the Tsinghua University Initiative Scientific Research Program in China (grant no. 523006011), and the funding from State Key Laboratory of Tribology in Advanced Equipment, 10.13039/501100004147Tsinghua University in China (grant no. SKLT2022C15).

## Author contributions

Conceptualization, Y.L., S.Y., Z.C., and X.L.; investigation, Y.L., S.Y., K.X., and L.J.; formal analysis, Y.L., S.Y., K.X., and L.J.; methodology, Y.L., S.Y., K.X., and Z.C.; visualization, Y.L., S.Y., and K.X.; writing – original draft, Y.L., S.Y., and Z.C.; writing – review and editing, Y.L., S.Y., and Z.C.; resources, Z.C. and X.L.; supervision, Z.C. and X.L.; funding acquisition and project administration, Z.C.

## Declaration of interests

The authors declare no competing interests.

## STAR★Methods

### Key resources table

**Table:** 

REAGENT or RESOURCE	SOURCE	IDENTIFIER
**Chemicals, peptides, and recombinant proteins**		

Sodium alginate	Aladdin	CAS: 9005-38-3
Eutectic gallium-indium	Aladdin	CAS: MFCD00144387
Polydimethysiloxane	Dow Corning	Sylgard 184
Polymide	Heshida	A01
Polyurethane	Yinuoweixun	1030D
Release paper	Oumai Technology	N/A
Nitrogen gas	Air Liquide	CAS 7727-37-9
Copper wire	Zhefei	RV-1
Silver paste	Jinshida	K-818

**Software and algorithms**		

Microsoft Excel	Microsoft	www.microsoft.com
Python	Python Software Foundation	www.python.org

**Other**		

Ultrasonic probe	Jiemeng	SN-P900
Ultraviolet lamp-172 nm	Cygnus	π-photonics
Dip-coating apparatus	Sanyan	SYDC-1
Spin coater	Setcas	KW-4A
Hotplate	DLAB	HP550-S
Optical microscope	Puzhe	XJ 52C
Transmission electron microscopy	JEOL	JEM2010
Scanning electron microscopy	Zeptools	Hitachi SU8010
Zeta potential analyzer	Malvern	Zetasizer Nano ZS
Rheometer	Anton Paar	MCR301
Contact angle goniometer	Dataphysics	OCAH200
Tensile testing machine	Sithai	ST-D500
Semiconductor parameter analyzer	Keysight	B1500A

### Experimental model and study participant details

All human participant experiments were conducted with one healthy adult female volunteer aged 26 years of the Han ethnicity. Age and gender have little influence on the detection of the sensor. The study involved non-invasive motion monitoring using stretchable strain sensors attached to skin over the elbow, wrist, finger, and knee for joint bending signals, as well as surface EMG electrode arrays placed on the forearm for gesture recognition. The participant performed repetitive joint bending at multiple angles and six predefined hand gestures following the same standardized protocol. No personal identifying information was collected, and no medical intervention was involved. No animals, plants, microorganisms, cell lines, or primary cell cultures were used in this work.

### Method details

#### Materials

Sodium alginate and eutectic gallium-indium (≥99.99%, 75.5 wt% of gallium and 24.5 wt% of indium) were purchased from Aladdin. Polydimethylsiloxane (Sylgard 184) was sourced from Dow Corning Co., Ltd. Polyimide substrates were supplied by Heshida Co., Ltd., while polyurethane substrates were provided by Yinuoweixun (Beijing) Optoelectronic Technology Co., Ltd. Release papers were acquired from Oumai Technology Co., Ltd.

#### Preparation of LM dispersion

Initially, 0.1 g of sodium alginate was dissolved in 20 mL of deionized water and stirred continuously for 1 hour. Subsequently, 1 g of LM was added into the solution. The mixture was then subjected to ultrasonic treatment for 90 minutes (SN-P900, Guangdong Jiemeng Ultrasonic Industry Co., Ltd.), which was operated at a power of 900 W with alternating cycles of 4 seconds on and 3 seconds off.

#### LM patterning

First, hydrophobic substrates (PDMS, PI, PU, and release paper) were masked with patterned chrome photo masks. These substrates were then exposed to a vacuum ultraviolet lamp (Cygnus Photonics, *π*-photonics) with a wavelength of 172 nm and an emission intensity of 10 ± 2 mW cm^-2^ under a nitrogen flow rate of 15 L min^-1^ to enhance the hydrophilicity of the exposed regions. The exposure durations were specifically set to 5 minutes for PDMS, 10 minutes for PI, 15 minutes for PU, and 15 minutes for the release paper. Next, the modified substrates were fixed onto a dip-coating apparatus (SYDC-1, Sanyan Experimental Instrument Co., Ltd.). The assembly process involved immersing the substrates into the LM dispersion for 5 seconds, withdrawing them at a constant speed of 20 mm/s, and allowing them to dry for 90 seconds. This sequential dip-coating and drying cycle was repeated 20 times to ultimately yield LM patterns that correspond to the photomasks.

#### Fabrication of stretchable strain sensors

PDMS substrates were covered with line-shaped photomasks featuring widths of 0.5, 1, and 1.5 mm, which were subsequently subjected to UV modification and LM particle assembly as described above. Copper wires were affixed to both ends of the deposited LM lines using conductive silver paste, followed by a curing process at 120 °C for 30 minutes. To encapsulate the device, a PDMS mixture was prepared by mixing the base elastomer and curing agent at a mass ratio of 10:1, which was then degassed in a vacuum. This PDMS mixture was poured over the patterned lines with the attached copper wires and spin-coated at 600 rpm for 30 seconds using a spin coater (KW-4A, Beijing Setcas Electronic Co., Ltd.) to ensure a uniform coating over the pattern surface. Finally, the upper PDMS layer was cured by heating at 120 °C for 15 minutes to complete the fabrication of the encapsulated LM stretchable strain sensors.

#### Characterizations and measurements

The macroscopic morphologies of the LM patterns were observed using an optical microscope (XJ 52C, Puzhe Photoelectric). Transmission electron microscopy (TEM, JEM2010) was employed to characterize the microscopic morphology of the LM particles. The surface microstructures and corresponding elemental distributions were analyzed through scanning electron microscopy (SEM, Hitachi SU8010) equipped with an energy-dispersive X-ray spectroscopy (EDS) mapping system. A nanoparticle size and Zeta potential analyzer (Zetasizer Nano ZS) was utilized to determine the Zeta potentials of the LM particles. The surface wettability of the substrates was assessed by measuring the contact angles of 2 μL liquid droplets via a contact angle goniometer (OCAH200, Dataphysics). The electro-mechanical responses of the strain sensors were recorded using a computer-controlled motorized tensile testing machine (ST-D500, Sithai) synchronized with a semiconductor parameter analyzer (B1500A, Keysight).

### Quantification and statistical analysis

Quantitative results are presented as mean ± standard deviation (SD); for figures without error bars, raw data are shown without transformation. Error bars in all figures represent SD. For Zeta potential values (Figures 1G and 1H), SNR measurements (Figures 4C and 4D), and resistance values (Figures S5 and S6), *n* represents the number of independent replicate measurements. For CNN-based gesture recognition, the dataset was split into 80% training and 20% testing, and the reported accuracy (94%) was calculated from the test set. Data analysis was performed using Microsoft Excel and Python.
