# Supplementary material for: Surface energy-directed assembly of liquid metal particles for intelligent wearable electronics
Source: iScience. 2026 Sep 25;29(10):117629. doi: 10.1016/j.isci.2026.117629 (PMC13635518; doi:10.1016/j.isci.2026.117629)
Supplement: Supplementary file 1 [file mmc1.pdf]

iScience, Volume *29*

Supplemental information

Surface energy-directed assembly of liquid metal particles for intelligent wearable electronics

Yubing Liu, Siqing Yuan, Kaiyi Xu, Linquan Jiang, Zhimin Chai, and Xinchun Lu

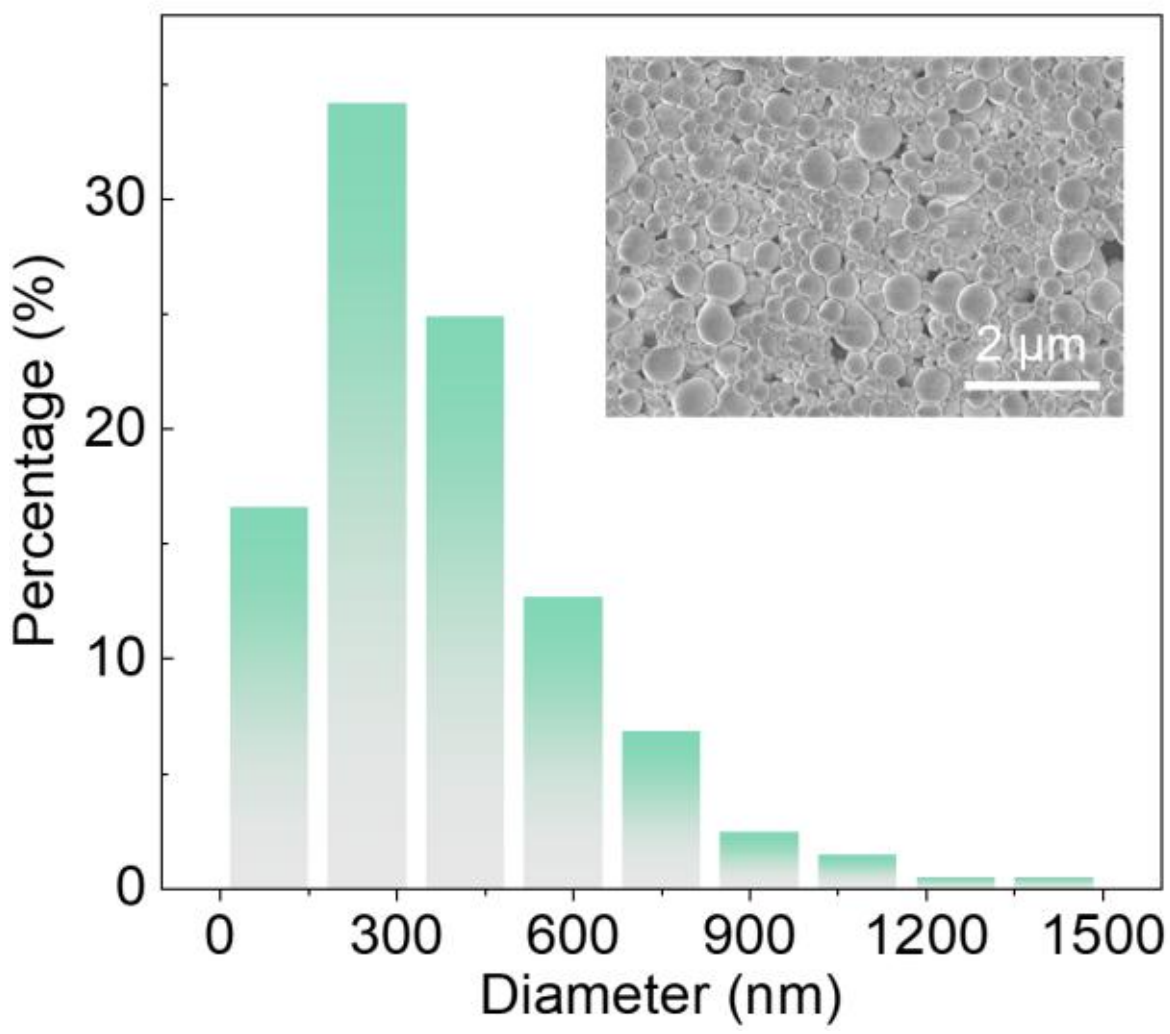

Figure S1. Statistical particle size distributions of LM dispersions after 60 minutes. Scale bar, 2 μm.

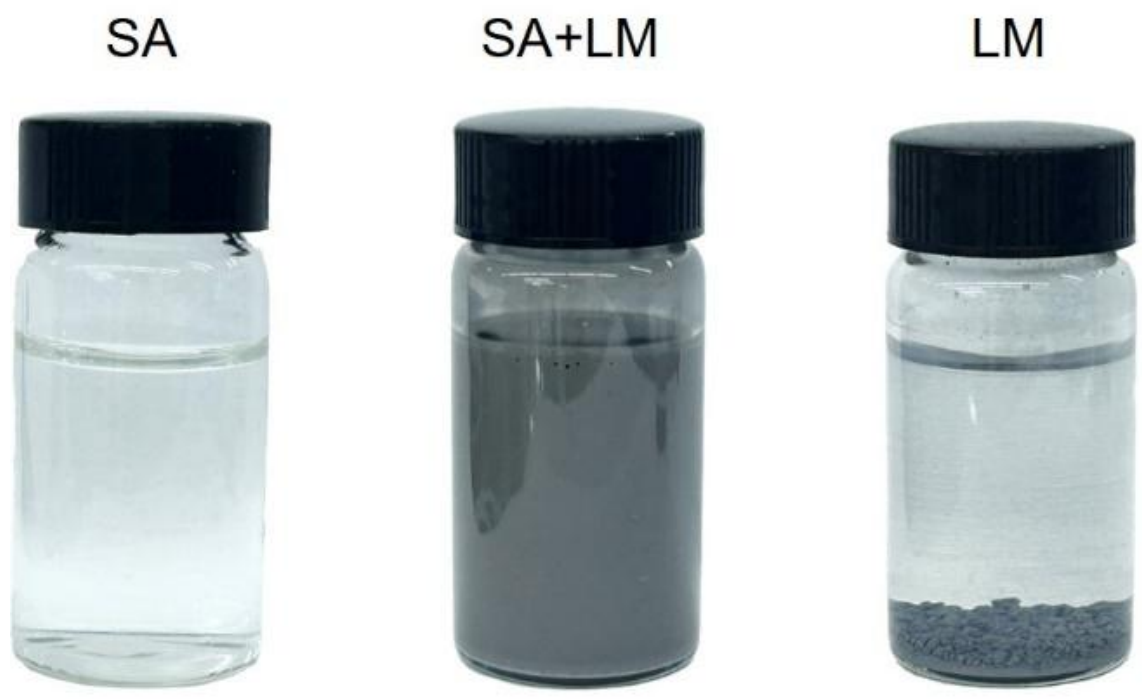

Figure S2. Photographs of three types of ink after 10 minutes of standing.

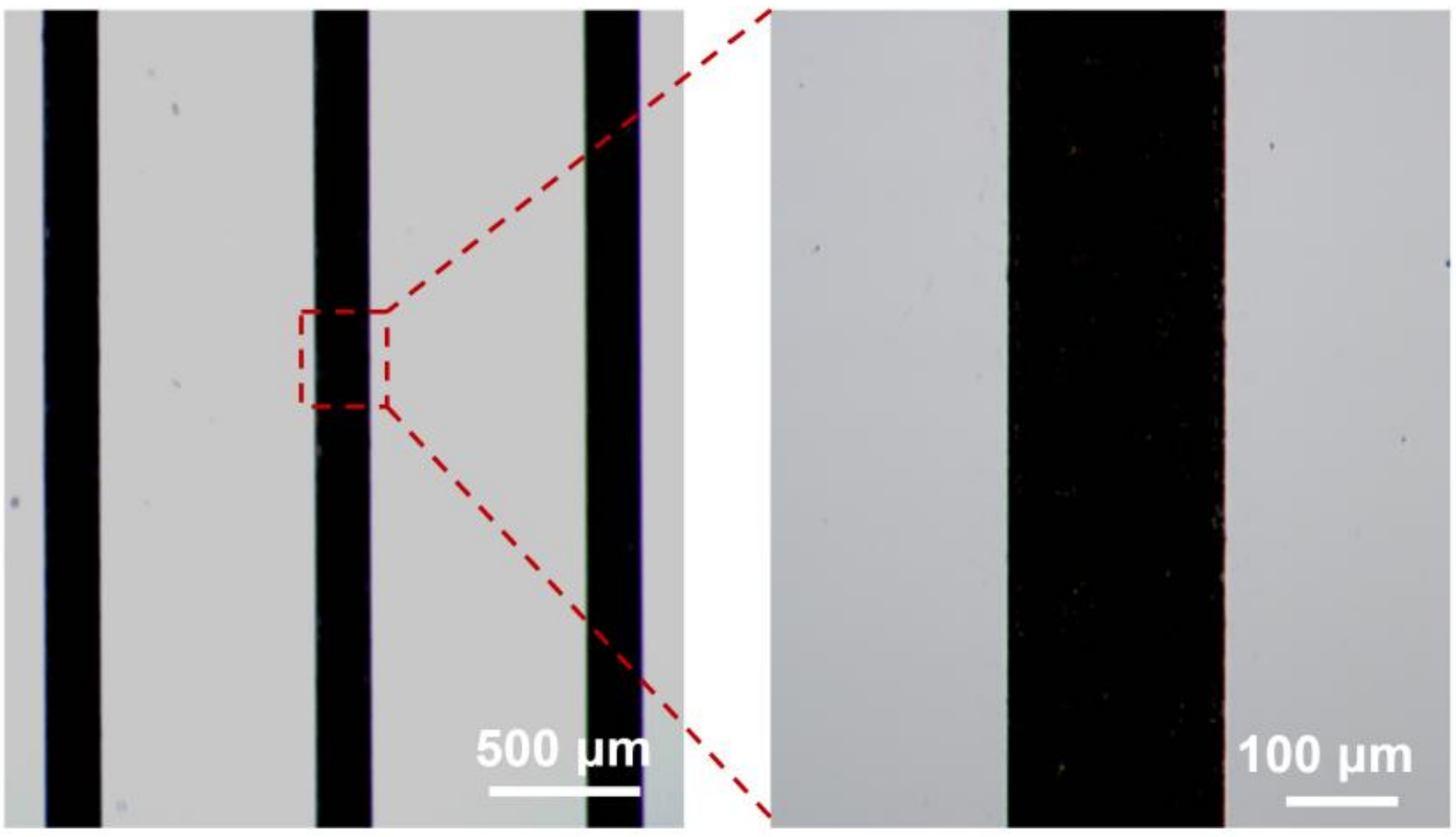

Figure S3. Optical microscope images for the LM line patterns with 200 μm of width. Scale bars, 500 μm (left) and 100 μm (right).

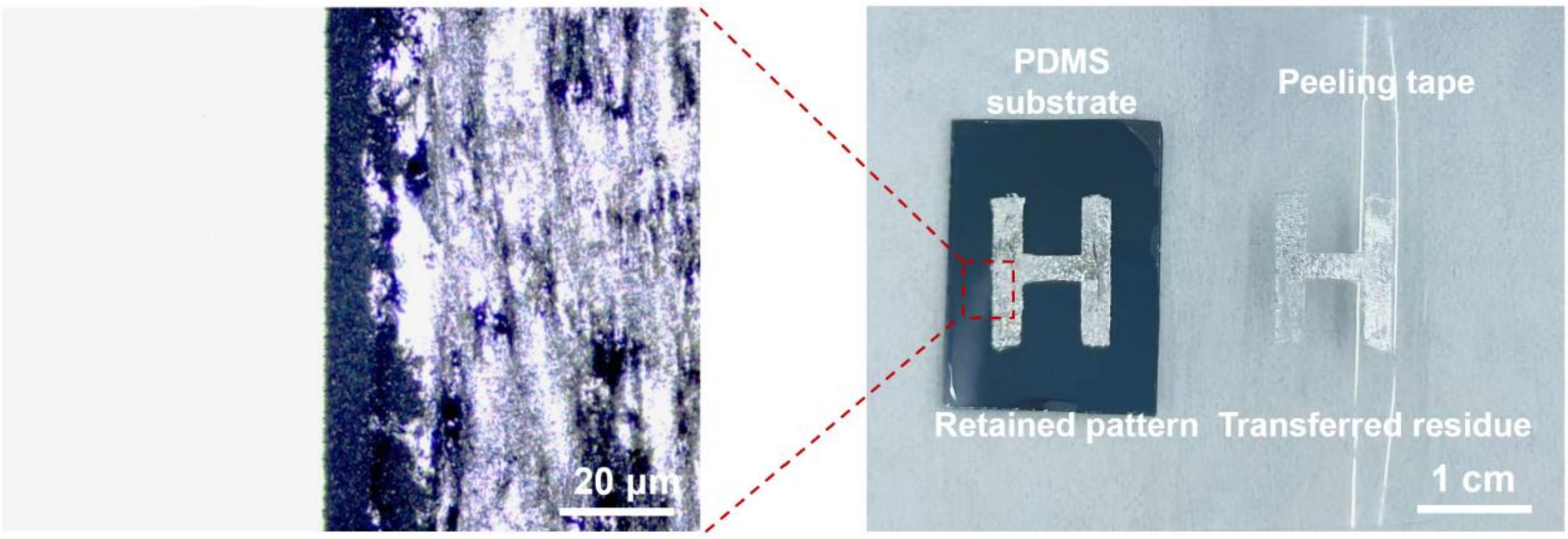

Figure S4. Tape peeling test of the LM pattern on PDMS. Scale bars, 20 μm (left) and 1 cm (right).

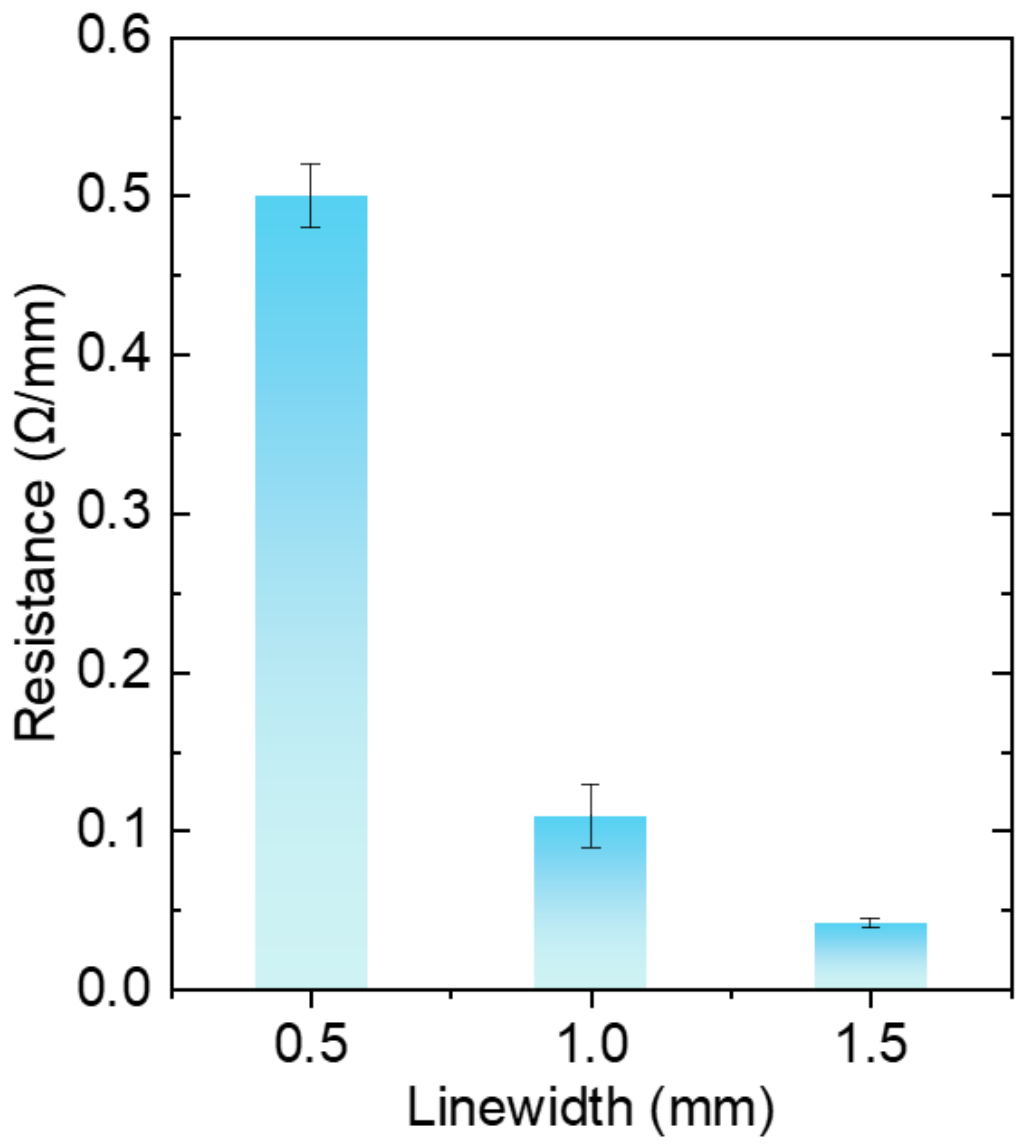

Figure S5. Original resistance of three strain sensor with different linewidths. Data are represented as mean ± SD (*n* = 3).

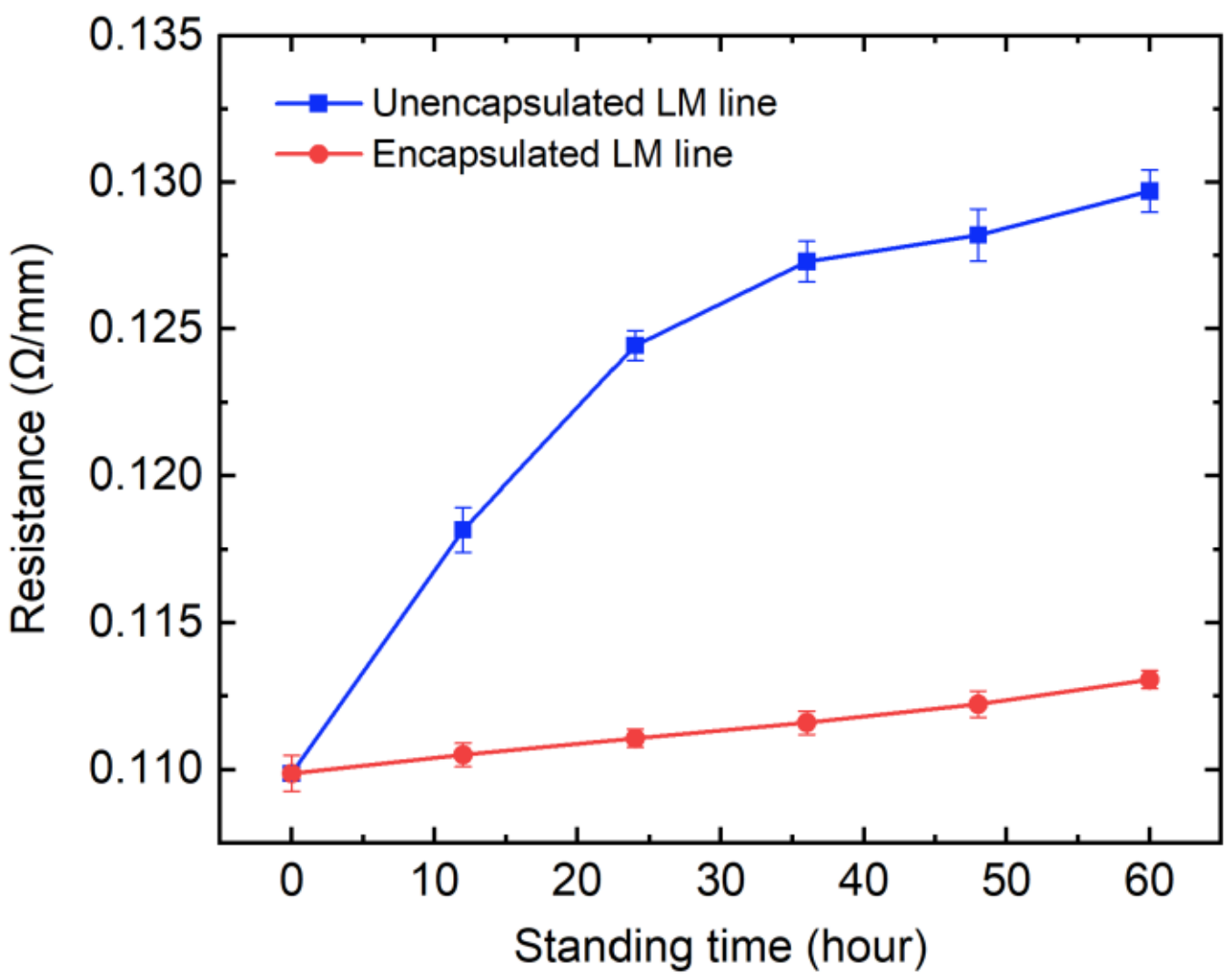

Figure S6. Resistance variation curves with increasing standing time of encapsulated and unencapsulated LM line with 1 mm of width. Data are represented as mean ± SD (*n* = 3).

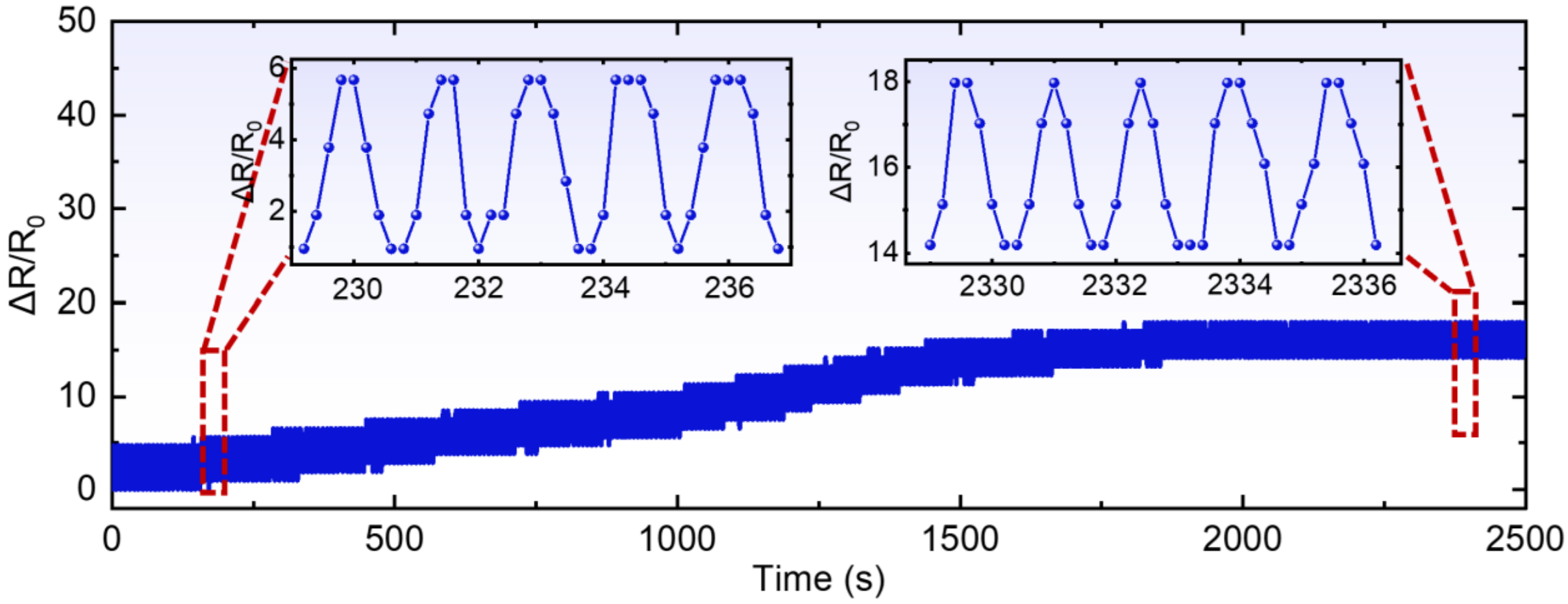

Figure S7. Long-term stability test for the strain sensor with 0.5 mm of linewidth.

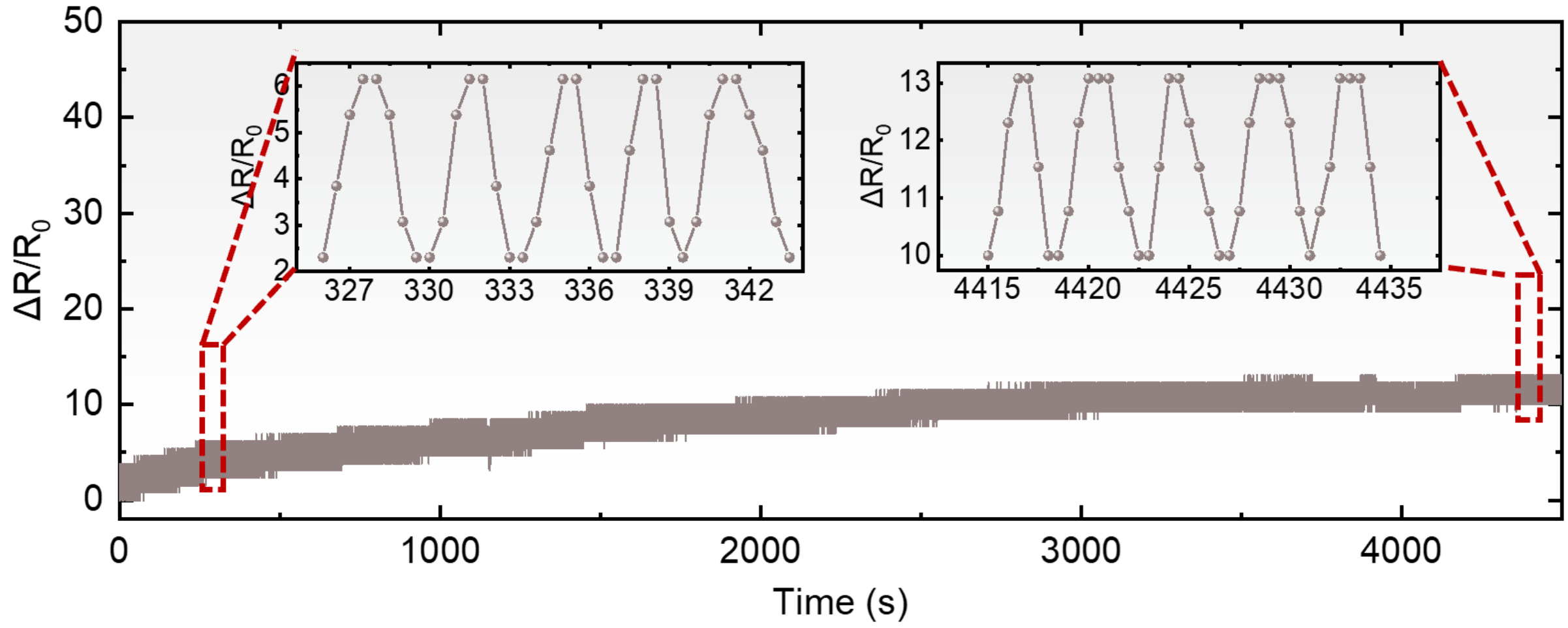

Figure S8. Long-term stability test for the strain sensor with 1 mm of linewidth.

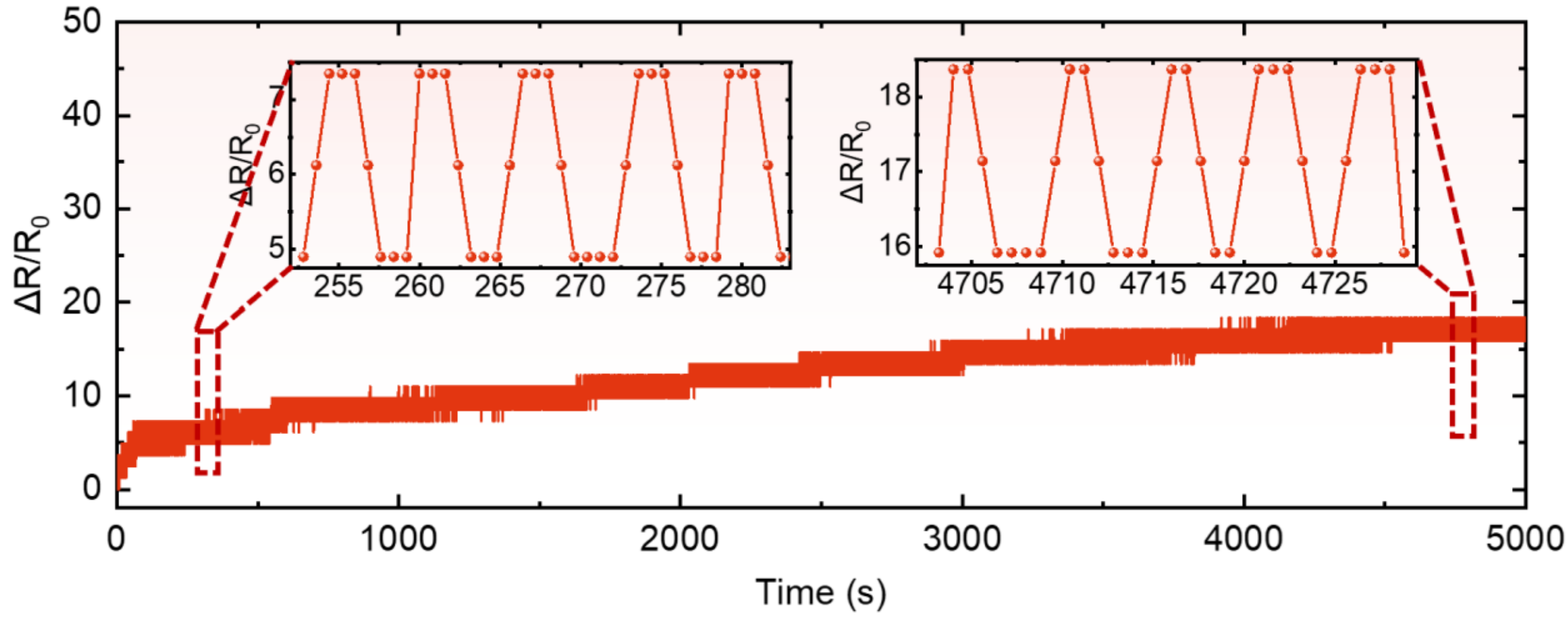

Figure S9. Long-term stability test for the strain sensor with 1.5 mm of linewidth.

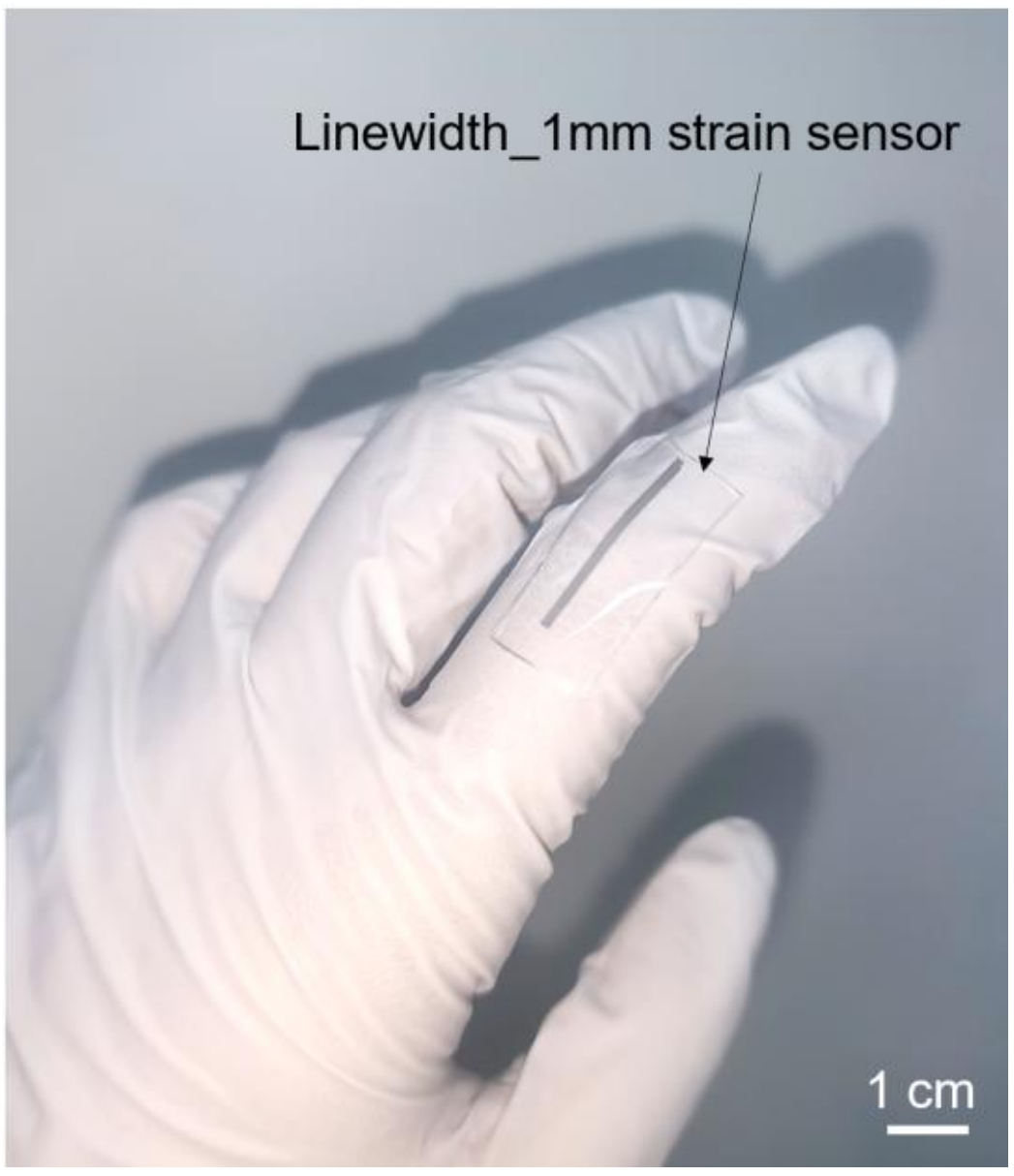

Figure S10. Photograph of the strain sensor with 1 mm of linewidth attaching to the human finger joint. Scale bar, 1 cm.

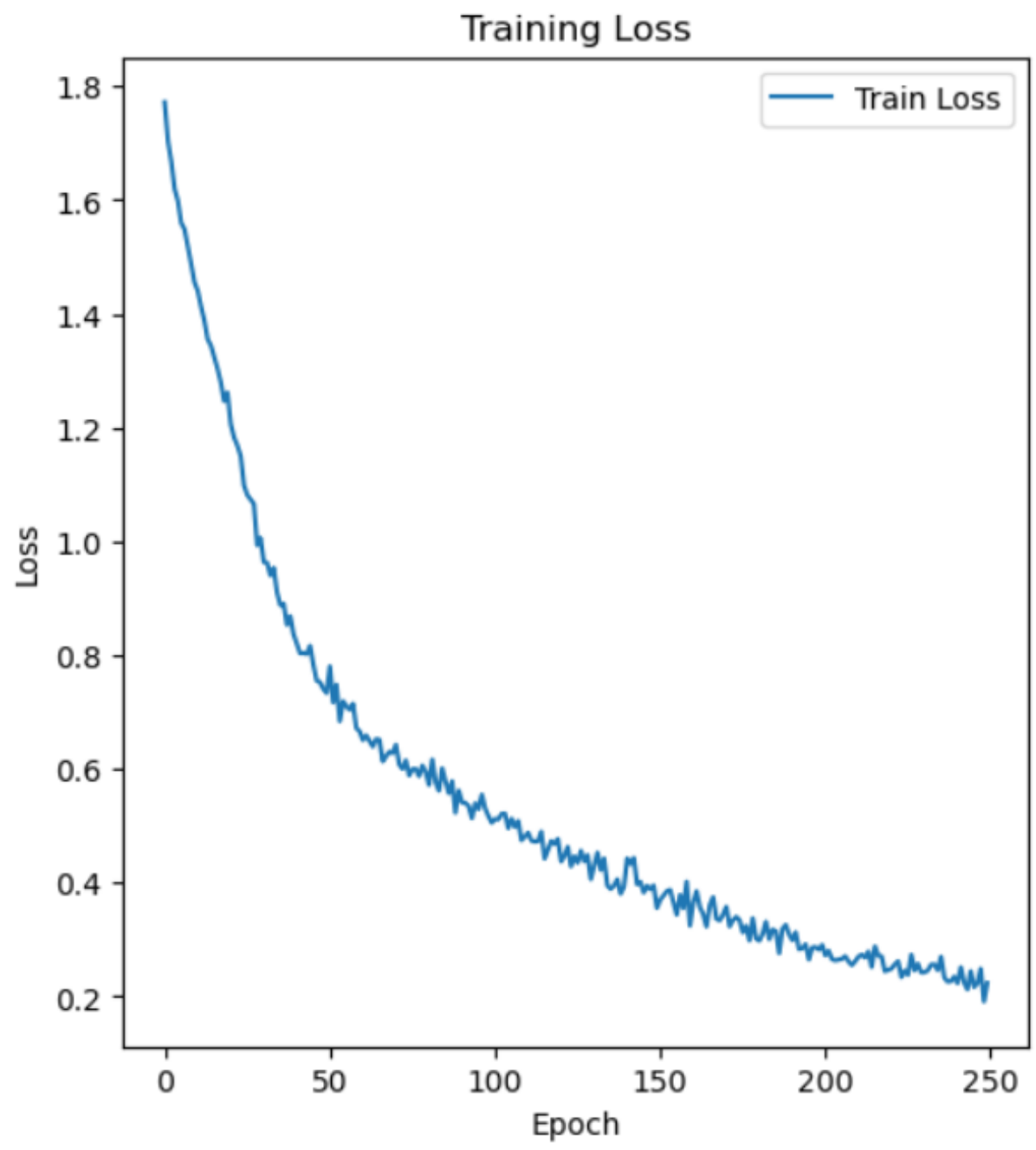

Figure S11. Training loss curve under 250 training cycles.
